# P-436. Epidemiology of Pediatric Invasive Pneumococcal Disease in an Inner-City Tertiary Care Center: A 13-Year Study and Post-Pandemic Insights

**DOI:** 10.1093/ofid/ofaf695.652

**Published:** 2026-01-11

**Authors:** Sarah Habbal, Nahed M Abdel-Haq, Ronald Thomas, Jocelyn Y Ang

**Affiliations:** Children's hospital of Michigan, Rockford, IL; Children's Hospital of Michigan. Central Michigan University, Detroit, MI; Central Michigan University, Detroit, Michigan; Wayne State University School of Medicine, Detroit, Michigan

## Abstract

**Background:**

Invasive pneumococcal disease (IPD) remains a significant cause of morbidity and mortality in children. While global IPD incidence declined during the COVID-19 pandemic due to mitigation measures, a resurgence followed the relaxation of these interventions. The primary objective is to describe the epidemiology of IPD at Children hospital, Detroit before and after the COVID-19 pandemic, including clinical manifestations, microbiology, treatment, and clinical outcomes.

**Methods:**

A retrospective chart review of IPD patients ≤21 years of age admitted between January 2010 & February 2024. Patients were categorized into 2 groups: pre-pandemic (2016-2020) and pandemic/post-pandemic (2020-2024)

**Results:**

143 cases were included. IPD incidence decreased during the pandemic (2020-2021) but rebounded in 2022, increasing from 0.6-0.8 to 1.7 per 1,000 admissions. Most cases (66.4%) were ≤ 5 years; 88 (61.5%) were males. 77 (53.8%) were previously healthy and 10 (6.9%) had sickle cell disease. Bacteremia (n=117, 82%) and complicated pneumonia (n=37, 25.9%) were the most common presentations. High susceptibility was observed for ceftriaxone (n=95/98, 97%) and penicillin (n=90/100, 90%) in non-meningitis isolates. However, susceptibilities were lower for ceftriaxone (18/22, 82%) and penicillin (15/22, 68%) in meningitis isolates.

Pneumococcal serotyping was performed in 11/143 (7.7%) cases; 63.6% were non PCV-13 vaccine serotypes. Low pneumococcal titers were found in 19/37 (51%) tested; 15/19 (79 %) were fully immunized. Titers for PCV7/PCV13 vaccine serotypes were low despite vaccinations.

No statistically significant differences were observed between the two periods regarding age, gender, or clinical presentation (p= >0.05). However, the proportion of unimmunized children increased significantly in the post-pandemic period (n=8/43, 18.6%) compared to pre-pandemic period (n=2/49, 4.1%; p=0.04).

**Conclusion:**

IPD incidence increased significantly post-pandemic, potentially due to decreased vaccination rates during the pandemic/post pandemic period. Recognizing these epidemiological shifts and addressing vaccination gaps with use of updated vaccine are critical to preventing future IPD outbreaks.

**Disclosures:**

Jocelyn Y. Ang, MD, astellas: Honoraria|astellas: speaker bureau|Eli Lilly and Company: Grant/Research Support|F. Hoffmann-La Roche Ltd: Grant/Research Support|Pfizer, Inc.: Honoraria

